# Influence of oral anticoagulation on success rates and risk of bleeding events after iStent inject implantation combined with phacoemulsification

**DOI:** 10.1007/s00417-020-04894-3

**Published:** 2020-08-20

**Authors:** Randolf A. Widder, Alexandra Lappas, Corinna Rennings, Matthias Hild, Gernot F. Roessler, Thomas S. Dietlein

**Affiliations:** 1 Department of Ophthalmology, St. Martinus-Krankenhaus Düsseldorf, Gladbacher Strasse 26, 40219 Düsseldorf, Germany; 2 Center of Ophthalmology, University of Cologne, Kerpener Strasse 62, 50937 Köln, Germany; 3 Department of Ophthalmology, RWTH Aachen, Pauwelsstrasse 30, 52074 Aachen, Germany

**Keywords:** Glaucoma, Glaucoma surgery, MIGS, Anticoagulation therapy, iStent

## Abstract

**Purpose:**

We conducted a retrospective study to evaluate the intraocular pressure (IOP) lowering effect, the success rates, and the risk of bleeding events of patients receiving an iStent inject combined with phacoemulsification under anticoagulation therapy compared with a matched control group.

**Methods:**

In this retrospective study, sixty-four eyes underwent an iStent inject implantation combined with phacoemulsification at two centers. Thirty-two eyes received surgery while under anticoagulation therapy, and another thirty-two eyes served as a control group matched for visual acuity, IOP, and medication score. Success was defined as criteria A and B (IOP < 18/21 mmHg, > 20% IOP reduction, no resurgery) and criteria C (IOP ≤ 15 mmHg, IOP reduction ≥ 40%, no resurgery). The clinical goal of the study was to determine the difference between the study and control groups with respect to IOP, medication score, and the frequency of intraoperative and postoperative bleeding events.

**Results:**

After a mean follow-up time of 1 year, the IOP lowered 28% from 20.1 ± 4.8 to 14.5 ± 3.7 mmHg in the group of 64 eyes. The medication score lowered 38% from 2.1 ± 1.1 to 1.3 ± 1.2. The two groups with and without anticoagulant agents did not significantly differ in postoperative IOP, medication score, success rates, or number of bleeding events.

**Conclusion:**

We conclude that in cataract surgery combined with the iStent inject a discontinuation of anticoagulant agents might not be necessary. It might be a good option in glaucoma surgery when anticoagulation treatment should not be interrupted and the target pressure is not very low.

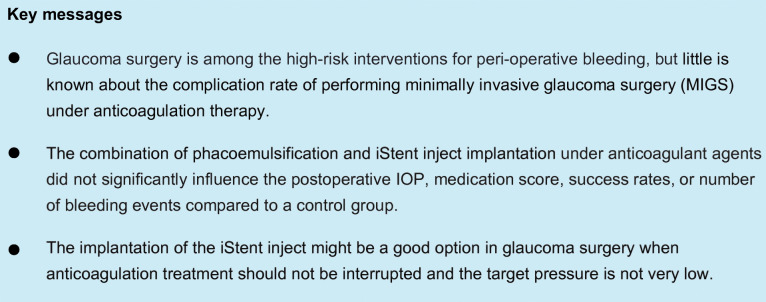



## Introduction

Glaucoma is a disease of the elder population where anticoagulant agents are used frequently. While it is generally accepted that intraocular bleeding after standard phacoemulsification cataract surgery does not justify the withdrawal of anticoagulant agents, there is no clear guidance for glaucoma surgery [1–4]. For trabeculectomy, Cobb et al. recommended the discontinuation of warfarin [5].

Little is known about the complication rate of performing minimally invasive glaucoma surgery (MIGS) under anticoagulation therapy. Surgery on angle structures may lead to intracameral bleeding by traumatizing angle tissue and by reflux bleeding through Schlemm’s canal [3]. Therefore, procedures with minimal angle trauma and significant efficacy in lowering intraocular pressure (IOP) are required.

The iStent inject (Glaukos Corp., Laguna Hills, CA, USA) might be a minimally invasive option and decrease the risk of intraocular bleeding. Therefore, we conducted a retrospective study to evaluate the IOP-lowering effect, the success rates, and the bleeding risk of patients receiving an iStent inject under anticoagulation therapy as compared with a matched control group.

## Materials and methods

This is a retrospective study. We found 147 eyes with open-angle or exfoliation glaucoma undergoing an iStent inject implantation combined with phacoemulsification in two centers (Cologne, Düsseldorf). Among these patients, there were 44 identified being under oral anticoagulation therapy. Six of these patients did not have a follow-up at all, and another 6 had a follow-up of less than 3 months. These eyes were excluded from further analysis. None of these patients had resurgery. Therefore, we included 32 eyes of 23 patients while patients were under oral phenprocoumon (20 eyes, Marcumar, Meda Pharma, Bad Homburg, Germany), apixaban (5 eyes, Eliquis, Bristol Myers-Squibb/Pfizer Pharma, München, Germany), rivaroxaban (5 eyes, Xarelto, Bayer Leverkusen, Germany), and dabigatran (2 eyes, Pradaxa, Boehringer Pharma, Ingelheim, Germany). The patients were advised to continue their anticoagulation therapy during surgery and postoperatively. None of these patients was under an additional anti-platelet therapy.

A control group without anticoagulation therapy or anti-platelet therapy receiving the same combined surgery was matched for preoperative IOP, preoperative medication score, and preoperative visual acuity (Table 1).

**Table 1:** Preoperative values

	Control group	Study group	*p*
Age (years)	74 ± 8	77 ± 6	0.06
Maximum preoperative IOP (mmHg)	27.0 ± 7.4	25.0 ± 6.8	0.44
Actual preoperative IOP (mmHg)	20.2 ± 3.9	20.1 ± 5.6	0.92
Medication score initial	2.1 ± 0.8	2.2 ± 1.3	0.90
Exfoliation glaucoma	*n* = 6	*n* = 7	0.76
Cup to disk ratio	0.7 ± 0.2	0.8 ± 0.2	0.19
Follow-up time (months)	12.5 ± 9.4	12.5 ± 8.6	0.97
Visual acuity preoperative (logMAR)	0.32 ± 0.26	0.32 ± 0.25	0.79

*IOP* intraocular pressure, *SD* standard deviation. The *p* value was calculated by the Mann-Whitney *U* test

All eyes underwent combined surgery with phacoemulsification, an implantation of an intraocular lens, and an implantation of two iStent inject stents. The first step of the surgery was a temporal 1.6-mm corneal incision. The anterior chamber was filled with a viscoelastic agent. The head of the patient was turned in the nasal direction while the microscope was tilted backwards so that the trabecular meshwork was visible through a modified Swan-Jacob lens. The trabecular meshwork was identified and two iStent inject stents were placed in the nasal part of Schlemm’s canal. Then, the corneal incision was widened to 2.8 mm and a standard phacoemulsification with two paracenteses at the 6 and 12 o’clock positions was performed. After insertion of the lens, the viscoelastic agent was removed, and a final check of the stent position was made by gonioscopy. Best corrected visual acuity was measured with standard Snellen charts and transformed into logMAR values. Intraocular pressure was measured by Goldmann applanation tonometry. For the preoperative values, the mean value of the last three examinations was used if available. At each examination during follow-up, gonioscopy was performed in all patients. To measure the success rates, we used different criteria: criteria A (IOP < 18 mmHg, > 20% IOP reduction, no resurgery), criteria B (IOP < 21 mmHg, > 20% IOP reduction, no resurgery), and criteria C (IOP ≤ 15 mmHg, ≥ 40% IOP reduction, no resurgery). Criteria A and B were chosen according to the Tube versus Trabeculectomy Study; criteria C was chosen according to the criteria of the World Glaucoma Association [6, 7].

The study and data accumulation were carried out with the approval of the Institutional Review Board (KK-0180, Ethik und Kommission Klinische Studien, Dernbacher Gruppe Katharina Kasper, Germany). The study was in adherence to the tenets of the Declaration of Helsinki and informed consent was obtained from all patients.

Statistical analysis was carried out using the Mann-Whitney *U* test, Fisher’s exact test, and log rank tests using the software package GraphPad Prism 6.0 (GraphPad Software Inc., San Diego, CA, USA). A *p* value < 0.05 was regarded as statistically significant. The clinical objective of the study was the difference of the postoperative IOP and the medication score as well as the frequency of intraoperative and postoperative bleeding events between the study and the control group at the end of the follow-up time.

## Results

**Fig. 1:**
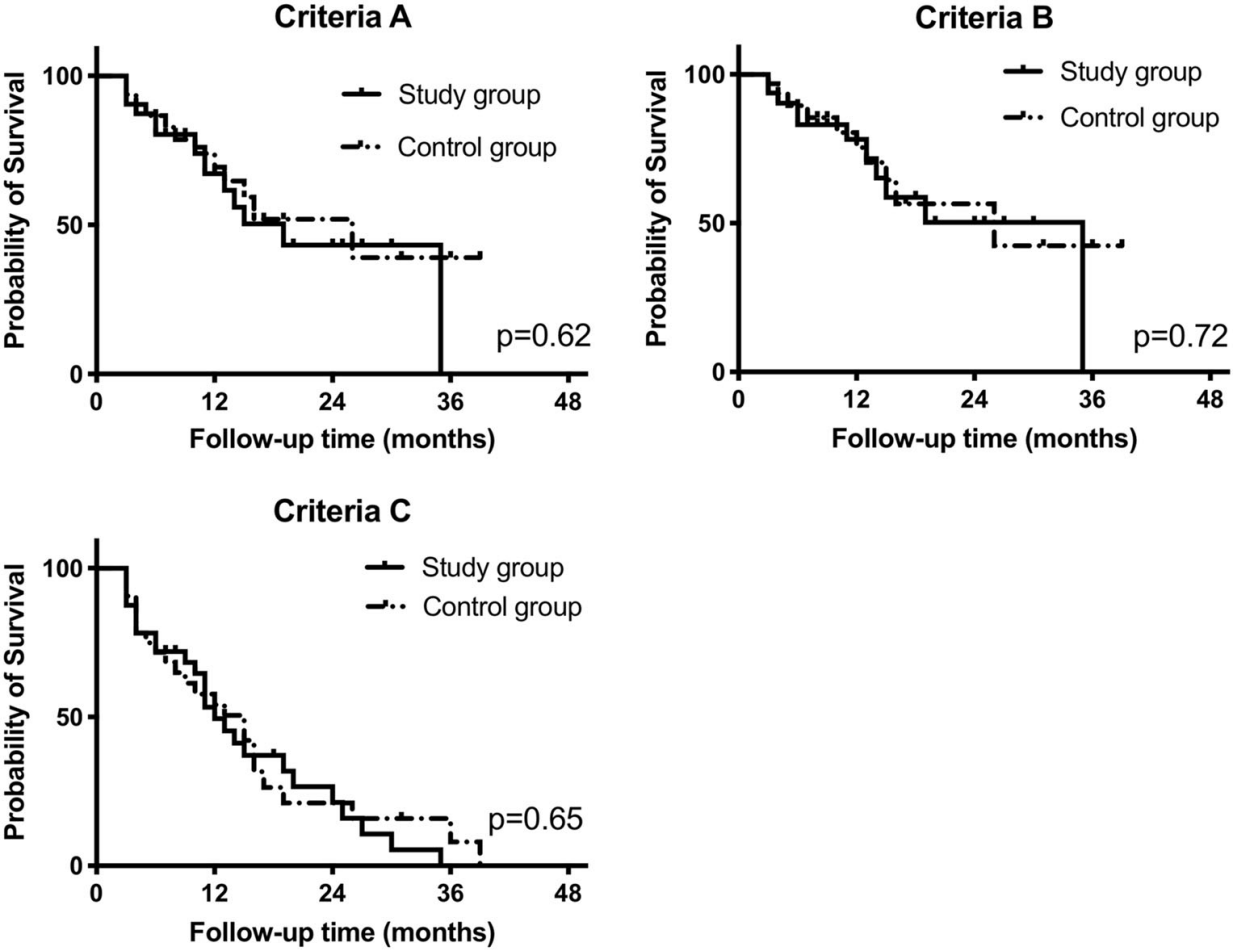
Success rates: Criteria A = IOP at follow-up < 18 mmHg, IOP reduction > 20%, no resurgery; Criteria B = IOP < 21 mmHg, > 20% reduction, no resurgery; Criteria C = IOP ≤ 15 mmHg, ≥ 40% reduction, no resurgery. The *p* value was calculated by the log rank test

After an average follow-up of 1 year, IOP was lowered from 20.1 ± 4.8 to 14.5 ± 3.7 mmHg in the total group of 64 eyes. This was a reduction of 28%. The medication score was lowered from 2.1 ± 1.1 to 1.3 ± 1.2 mmHg, which was a reduction of 38%. The two groups with anticoagulant agents (study group) and without anticoagulant agents (control group) did not differ significantly in postoperative IOP, postoperative medication score, or success rates (Table 2; Fig. 1).

**Table 2:** Intraocular pressure (IOP) and medication score (Med) before surgery, 1 day after surgery, and at the end of follow-up. The *p* value was calculated by the Mann-Whitney *U* test

	All	Control group	Study group	*p*
IOP preoperative (mmHg)	20.1 ± 4.8	20.2 ± 3.9	20.1 ± 5.6	0.92
IOP day 1 (mmHg)	14.8 ± 8.0	15.3 ± 7.8	14.3 ± 8.2	0.33
IOP follow-up (mmHg)	14.5 ± 3.7	14.5 ± 3.8	14.5 ± 3.7	0.92
Med preoperative	2.1 ± 1.1	2.1 ± 0.8	2.2 ± 1.3	0.90
Med follow-up	1.3 ± 1.2	1.4 ± 1.2	1.2 ± 1.1	0.47

On the day after surgery, there was 1 patient in the study group under phenprocoumon showing a 1-mm hyphema. The IOP was 10 mmHg. The hyphema vanished the next day. No other postoperative bleeding events were reported. Therefore, the rate of postoperative bleeding events was 1/32 vs. 0/32 (*p* = 1, Fisher’s exact test). Side effects like a flat anterior chamber, choroidal effusion, leakage of aqueous humor, endophthalmitis, or retinal ablation were not found. Macular edema was found in 1 eye of the study group and in 1 eye of the control group.

## Discussion

We addressed whether glaucoma patients under anticoagulant agents are at a higher bleeding risk, and whether that would indicate that these patients should discontinue their medication before surgery.

In the 32 patients under anticoagulant agents, we did not find postoperative bleeding events except by one eye when a transient bleeding in the chamber angle with hyphema was found 1 day after surgery. This patient did not show a higher IOP, and the hyphema disappeared the next day. Therefore, the conclusion from our data is that the implantation of the iStent inject under anticoagulant agents does not lead to clinically relevant bleeding events.

Another concern might be that blood could obstruct the stents and lead to a worse outcome compared with patients who are not under anticoagulant agents. Therefore, we compared the eyes in our study with those of a control group without anticoagulant agents. We found no difference between the anticoagulation group and the control group with respect to postoperative IOP, reduction of medication score, and success rates. There was no significant or clinically relevant difference between the two groups.

Glaucoma surgery is among the high-risk interventions for peri-operative bleeding [4]. Cobb et al. found that patients under warfarin or aspirin experienced a higher rate of intraocular bleeding after trabeculectomy [5]. A hyphema occurred in 55% of the eyes under anti-platelet therapy and anticoagulation therapy as compared with 28% without therapy. In patients on aspirin, this did not have an impact on the surgical outcome, but a hyphema associated with warfarin led to a failure in 4 out of 5 patients. Therefore, the authors recommended the discontinuation of warfarin before a trabeculectomy.

Little is known about the complication rate of performing MIGS under anticoagulation therapy. The iStent inject uses the same pathway to lower IOP as trabectome surgery. Both techniques aim to bypass the trabecular meshwork to increase the aqueous outflow through Schlemm’s canal. Surgery on angle structures can lead to intracameral bleeding, by traumatizing angle tissue, and reflux bleeding from Schlemm’s canal [3]. Trabectome surgery causes a larger trauma to angle structures and is known to show transient intraocular bleeding during surgery in up to 100% of the eyes [8]. Surgery with the iStent inject also leads to intraocular bleeding but to a lesser extent. Salimi et al. described a transient hyphema in 5% of their patients, while Hengerer et al. described one patient with transient hyphema among 44 cases [9, 10]. Usually the hyphema after the implantation of an iStent inject is transient and vanishes within a few days, but the appearance of intraocular bleeding events raises the question of whether patients under anticoagulation therapy are at a higher risk of intraocular bleeding.

Another concern is a late onset of intraocular bleeding into the anterior chamber, as has been described in patients 2–31 months after trabectome surgery [8]. Khouri et al., as well as Sandhu et al., describe a case of late onset and recurring bleeding after a single iStent insertion (not iStent inject) [11, 12]. While Khouri et al. detected a malposition of the iStent leading to bleeding events, Sandhu et al. concluded that the bleeding resulted from reflux from Schlemm’s canal into the anterior chamber. Both patients were not reported to be under anticoagulant agents.

Comparing the iStent inject with the single iStent, early intraocular bleeding was found after both techniques with a lower frequency after iStent inject [13]. Nevertheless, we know that bleeding events also happen in iStent inject surgery, probably less frequently than in trabectome surgery [14].

The results from our study suggest that in cataract surgery combined with the iStent inject a discontinuation of anticoagulant agents might not be necessary. It might be a good option in glaucoma surgery when anticoagulation treatment should not be interrupted and the target pressure is not very low. Shortcoming of the study is its retrospective nature and the small amount of patients related to the rare nature of bleeding events. For a definitive answer, a prospective study with more patients would be useful.
